# Supplementary material for: Structural basis for transcription complex disruption by the Mfd translocase
Source: eLife. 2021 Jan 22;10:e62117. doi: 10.7554/eLife.62117 (PMC7864632; doi:10.7554/eLife.62117)
Supplement: Supplementary file 1. [file elife-62117-supp1.docx]

**Supplementary file 1 | Cryo-EM data collection, refinement and validation statistics.**

|  |  | | | | | | |
| --- | --- | --- | --- | --- | --- | --- | --- |
| **Data collection and processing** |  | | | | | | |
| Voltage (kV) | 300 | | | | | | |
| Electron exposure (e–/Å^2^) | 60 | | | | | | |
| Defocus range (μm) | -0.8 to -2.4 | | | | | | |
| Pixel size (Å) | 1.3 | | | | | | |
| Symmetry imposed | C1 | | | | | | |
| Initial particle images (no.) |  | | | | | | |
| Structure | L1 | L2(adp) | C1(ATP) | C2(ATP) | C3(adp) | C4(ADP) | C5(ATP) |
| Final particle images (no.) | 13,013 | 16,067 | 23,856 | 85,840 | 47,312 | 66,617 | 102,741 |
| Map resolution (Å)  FSC threshold 0.143 | 4.1 | 4.0 | 3.9 | 3.5 | 4.0 | 3.6 | 3.4 |
| Map resolution range (Å) | 3.4 - 10 | 3.5 - 10 | 3.3 - 10 | 3.0 - 9 | 3.3 - 10 | 3.1 - 9.5 | 2.8 - 8 |
| EMDB accession code | EMD-21996 | EMD-22006 | EMD-22012 | EMD-22039 | EMD-22043 | EMD-22044 | EMD-22045 |
| **Refinement** |  |  |  |  |  |  |  |
| Model composition  Non-hydrogen atoms  Protein residues  Nucleic acid residues  Ligands | 35,968  4,314  113  3 (1 Mg^2+^,  2 Zn^2^) | 36,058  4,316  113  4 (1 Mg^2+^, 2 Zn^2+^, 1 ADP) | 36,078  4,316  113  4 (1 Mg^2+^,  2 Zn^2+^, 1 ATP) | 36,055  4,316  111  5 (2 Mg^2+^,  2 Zn^2+^, 1 ATP) | 36,019  4,316  112  4 (1 Mg^2+^,  2 Zn^2+^, 1 ADP) | 36,086  4,316  113  5 (2 Mg^2+^, 2 Zn^2+^, 1 ADP) | 36,075  4,315  113  5 (2 Mg^2+^, 2 Zn^2+^, 1 ATP) |
| *B* factors (Å^2^)  Protein  Nucleic acid  Ligands | 127.1  216.4  154.1 | 100.09  166.31  83.15 | 107.4  152.3  114.8 | 66.56  123.83  47.00 | 100.2  216.7  98.44 | 83.93  144.01  82.98 | 50.34  104.1  32.07 |
| R.m.s. deviations  Bond lengths (Å)  Bond angles (°) | 0.008  1.109 | 0.008  1.138 | 0.009  1.196 | 0.010  0.989 | 0.009  1.197 | 0.006  0.949 | 0.005  0.810 |
| Validation  MolProbity score  Clashscore  Poor rotamers (%) | 2.07  12.03  0.0 | 2.54  10.77  0.0 | 1.99  9.22  0.06 | 2.01  9.52  0.0 | 1.98  9.26  0.06 | 1.96  8.83  0.0 | 1.89  7.70  0.03 |
| Ramachandran plot^a^  Favored (%)  Allowed (%)  Outliers (%) | 92.29  7.69  0.02 | 92.24  7.73  0.02 | 91.71  8.27  0.02 | 90.41  9.52  0.07 | 92.04  7.92  0.05 | 91.97  7.99  0.05 | 92.36  7.59  0.05 |
| PDB accession code | 6X26 | 6X2F | 6X2N | 6X43 | 6X4W | 6X4Y | 6X50 |

^a^ Refinement/validation parameters as calculated by PHENIX real_space_refine (Adams et al., 2010) and MOLPROBITY (Chen et al., 2010).
