# Supplementary material for: Structural basis for transcription complex disruption by the Mfd translocase
Source: eLife. 2021 Jan 22;10:e62117. doi: 10.7554/eLife.62117 (PMC7864632; doi:10.7554/eLife.62117)
Supplement: Supplementary file 2. [file elife-62117-supp2.docx]

**Supplementary file 2 | Bound nucleotide test.**

|  |  | average real space correlation coefficient^a^ | |  |
| --- | --- | --- | --- | --- |
| structure | nominal resolution  (Å) | nucleotide (+Mg^2+^ if modelled) + contact residues^b^ | | (avg_original_ - avg_test_)  [(avg_original_+avg_test_)/2]  (%) |
| L2(ADP) | 4.0 | avg_original_ | 0.7140 | -0.13 |
| L2(ATPtest) |  | avg_test_ | 0.7231 |  |
|  | | | | |
| C1(ATP) | 3.9 | avg_original_ | 0.8018 | 3.5 |
| C1(ADPtest) |  | avg_test_ | 0.7745 |  |
|  | | | | |
| C2(ATP) | 3.5 | avg_original_ | 0.9053 | 2.5 |
| C2(ADPtest) |  | avg_test_ | 0.8826 |  |
|  | | | | |
| C3(ADP) | 4.0 | avg_original_ | 0.8558 | -0.023 |
| C3(ATPtest) |  | avg_test_ | 0.8560 |  |
|  | | | | |
| C4(ADP) | 3.6 | avg_original_ | 0.8338 | 1.2 |
| C4(ATPtest) |  | avg_test_ | 0.8239 |  |
|  | | | | |
| C5(ATP) | 3.4 | avg_original_ | 0.9064 | 2.3 |
| C5(ADPtest) |  | avg_test_ | 0.8857 |  |

^a^Calculated using validation tools in PHENIX (Adams et al., 2010).

^b^The listed correlation coefficient is an average of the value for the nucleotide, the Mg^2+^-ion (if modelled), and the following Mfd residues that contact the nucleotide in at least one of the structures: F597, F599, E600, T602, Q605, D629, G631, F632, G633, K634, T635, E636, H665, D729, E730, P780, R783, G874, R902, R905.
