# Supplementary material for: Structural basis for transcription complex disruption by the Mfd translocase
Source: eLife. 2021 Jan 22;10:e62117. doi: 10.7554/eLife.62117 (PMC7864632; doi:10.7554/eLife.62117)
Supplement: Supplementary file 3. [file elife-62117-supp3.docx]

**Supplementary file 3. Conformational changes for the RNAP component of the Mfd-EC structures.**

|  | L2(adp) | | C1(ATP) | | C2(ATP) | | C3(adp) | | C4(ADP) | | C5(ATP) | |
| --- | --- | --- | --- | --- | --- | --- | --- | --- | --- | --- | --- | --- |
|  | align | rms_cur | align | rms_cur | align | rms_cur | align | rms_cur | align | rms_cur | align | rms_cur |
| L1(atp) | 0.556  (2798) | 0.82  (3171) | 0.596  (2832) | 0.877  (3172) | 0.661  (2536) | 1.46  (3169) | 0.707  (2362) | 3.638  (3164) | 0.642  (2318) | 2.203  (3171) | 0.553  (2583) | 2.188  (3172) |
| L2(adp) |  | | 0.456  (2860) | 0.585  (3171) | 0.586  (2409) | 1.318  (3168) | 0.711  (2322) | 3.61  (3163) | 0.603  (2332) | 2.244  (3170) | 0.565  (2625) | 2.313  (3171) |
| C1(ATP) |  | |  | | 0.544  (2393) | 1.24  (3169) | 0.693  (2279) | 3.514  (3164) | 0.548  (2328) | 2.177  (3171) | 0.522  (2592) | 2.3  (3172) |
| C2ATP) |  | |  | |  | | 0.546  (2343) | 2.69  (3167) | 0.303  (2480) | 1.818  (3168) | 0.346  (2319) | 2.244  (3169) |
| C3(adp) |  | |  | |  | |  | | 0.57  (2326) | 2.669  (3163) | 0.585  (2351) | 3.518  (3164) |
| C4(ADP) |  | |  | |  | |  | |  | | 0.393  (2492) | 1.044  (3171) |
