# Supplementary material for: Structural basis for transcription complex disruption by the Mfd translocase
Source: eLife. 2021 Jan 22;10:e62117. doi: 10.7554/eLife.62117 (PMC7864632; doi:10.7554/eLife.62117)
Supplement: Supplementary file 4. [file elife-62117-supp4.docx]

**Supplementary file 4. Conformational changes for the Mfd component of the Mfd-EC structures.**

|  | L1(adp) | | L2(adp) | | C1(ATP) | | C2(ATP) | | C3(adp) | | C4(ADP) | | C5(ATP) | |
| --- | --- | --- | --- | --- | --- | --- | --- | --- | --- | --- | --- | --- | --- | --- |
|  | align | rms_cur | align | rms_cur | align | rms_cur | align | rms_cur | align | rms_cur | align | rms_cur | align | rms_cur |
| 2EYQ | 4.998  (916) | 9.179  (1113) | 19.606  (889) | 34  (1106) | 12.002  (897) | 36.909  (1107) | 13.519  (907) | 37.193  (1115) | 18.508  (940) | 36.746  (1109) | 16.586  (928) | 35.991  (1109) | 10.253  (891) | 36.375  (1109) |
| L1(atp) |  | | 20.303  (910) | 32.199  (1115) | 13.454  (901) | 37.08  (1110) | 16.895  (930) | 36.536  (1114) | 14.825  (905) | 38.634  (1114) | 13.717  (900) | 37.494  (1112) | 12.331  (898) | 36.58  (1112) |
| L2(adp) |  | |  | | 20.605  (1032) | 26.011  (1135) | 22.779  (1075) | 25.75  (1131) | 20.421  (1076) | 23.088  (1139) | 21.076  (1140) | 21.205  (1139) | 24.73  (1140) | 24.818  (1135) |
| C1(ATP) |  | |  | |  | | 0.827  (930) | 3.954  (1134) | 3.686  (1020) | 5.674  (1138) | 4.714  (976) | 7.676  (1136) | 0.747  (925) | 7.109  (1136) |
| C2(ATP) |  | |  | |  | |  | | 3.888  (1031) | 5.171  (1136) | 4.449  (1003) | 7.294  (1134) | 0.667  (915) | 5.769  (1132) |
| C3(adp) |  | |  | |  | |  | |  | | 1.453  (953) | 5.61  (1140) | 3.637  (938) | 7.392  (1138) |
| C4(ADP) |  | |  | |  | |  | |  | |  | | 4.946  (1122) | 5.098  (1136) |
