# Supplementary material for: Structural basis for transcription complex disruption by the Mfd translocase
Source: eLife. 2021 Jan 22;10:e62117. doi: 10.7554/eLife.62117 (PMC7864632; doi:10.7554/eLife.62117)
Supplement: Supplementary file 5. [file elife-62117-supp5.docx]

**Supplementary file 5. Conformational changes for the entire Mfd-EC complexes.**

|  | L2(adp) | | C1(ATP) | | C2(ATP) | | C3(adp) | | C4(ADP) | | C5(ATP) | |
| --- | --- | --- | --- | --- | --- | --- | --- | --- | --- | --- | --- | --- |
|  | align | rms_cur | align | rms_cur | align | rms_cur | align | rms_cur | align | rms_cur | align | rms_cur |
| L1(atp) | 0.747  (3143) | 35.736  (4286) | 0.851  (3177) | 42.301  (4282) | 1.119  (3071) | 44.219  (4283) | 1.934  (3025) | 46.889  (4278) | 1.631  (3034) | 44.535  (4283) | 1.185  (3061) | 44.162  (4284) |
| L2(adp) |  | | 0.534  (3135) | 19.00  (4306) | 1.037  (3033) | 22.844  (4299) | 1.521  (2977) | 24.626  (4302) | 1.269  (3009) | 23.003  (4309) | 0.879  (3066) | 24.152  (4306) |
| C1(ATP) |  | |  | | 0.967  (3034) | 6.839  (4303) | 1.450  (2957) | 10.137  (4302) | 1.067  (2937) | 9.960  (4307) | 0.788  (3033) | 11.428  (4308) |
| C2(ATP) |  | |  | |  | | 2.127  (3247) | 5.428  (4303) | 0.458  (2928) | 4.699  (4302) | 0.793  (2926) | 7.738  (4301) |
| C3(adp) |  | |  | |  | |  | | 2.765  (4186) | 3.736  (4303) | 2.905  (3540) | 6.433  (4302) |
| C4(ADP) |  | |  | |  | |  | |  | | 0.633  (3071) | 4.491  (4307) |
