# Supplementary material for: Structural basis for transcription complex disruption by the Mfd translocase
Source: eLife. 2021 Jan 22;10:e62117. doi: 10.7554/eLife.62117 (PMC7864632; doi:10.7554/eLife.62117)
Supplement: Supplementary file 6. [file elife-62117-supp6.docx]

**Supplementary file 6. Conformational transitions (translation of center-of-gravity and rotation) for Mfd domains.**

|  | Structural transition | | | | | | | |
| --- | --- | --- | --- | --- | --- | --- | --- | --- |
|  | L(0) -> L1(atp) | | | L1(atp) -> L2(adp) | | L2(adp) -> C1(ATP) | |  |
| Mfd structural domains | cog translation (Å) | rotation (°) | cog translation (Å) | | rotation (°) | cog translation (Å) | rotation (°) |  |
| D1-D3 | 7.7 | 28.7 | 59.6 | | 162.0 | 44.5 | 102.0 |  |
| D4(RID) | 0.37 | 2.8 | 3.9 | | 11.0 | 0.7 | 1.6 |  |
| D5(TD1) | 10.9 | 42.5 | 64.5 | | 259.1 | 2.8 | 16.6 |  |
| D6(TD2) | 15.9 | 36.6 | 61.0 | | 256.7 | 2.7 | 10.0 |  |
| D7 | 4.3 | 24.4 | 81.8 | | 147.7 | 6.8 | 14.0 |  |
|  |  |  |  | |  |  |  |  |
| Mfd overall | cog translation (Å) | rotation (°) |  | |  |  |  |  |
| C1(ATP) -> C2(ATP) | 9.8 | 15.5 |  | |  |  |  |  |
| C2(ATP) -> C3(adp) | 7.9 | 14.8 |  | |  |  |  |  |
| C3(adp) -> C4(ADP) | 8.3 | 1.9 |  | |  |  |  |  |
| C4(ADP) -> C5(ATP) | 5.1 | 10.4 |  | |  |  |  |  |
| C5(ATP) -> C1(ATP) | 12.6 | 30.9 |  | |  |  |  |  |
